# On the scientific misconduct: a letter from Russia

**DOI:** 10.1590/S1679-45082013000100027

**Published:** 2013

**Authors:** Sergei Jargin

In an editorial on scientific misconduct, Glina points out two important issues: the pressure to publish for attainment of academic positions, and the difficulty of detecting fraud in scientific research^(1)^. The editorial agrees with our experience in Russia, where no articles have been retracted so far. During the 1990s, detection of scientific misconduct seemed to be easy: plagiarism was ample^(2)^, and some reports were at variance with the laws of physics^(3)^ or principles known in medical science^(4)^. Meanwhile, unscrupulous authors have perfected themselves in tangling and befogging the text, making evaluation of their papers increasingly difficult. Mutual cover-up is usual. Considering the “improvement” of fraudulent skills, scientists, editors, and authorities must jointly combat such fraudulence. Also, it is important to stress that whistleblowers must be protected from revenge or retaliation. In conclusion, response to scientific misconduct requires a national body to provide leadership and guidelines, and whistleblowers need a safe, confidential place to report scientific misconduct^(5)^.
